# Identification of improved signal peptides for heterologous expression in *Saccharomyces* using a screen that exploits *Gaussia* luciferase

**DOI:** 10.1038/s41598-025-09669-6

**Published:** 2025-07-04

**Authors:** Ginevra Camboni, Jared Cartwright, Gideon Grogan

**Affiliations:** 1 Department of Chemistry, University of York, Heslington, York, YO10 5DD UK; 2 Department of Biology, University of York, Heslington, York, YO10 5DD UK

**Keywords:** *Komagataella phaffii*, *Pichia pastoris*, Gaussia luciferase, Signal peptide, Unspecific peroxygenase, Directed evolution, Biotechnology, Molecular biology

## Abstract

A high-throughput and sensitive screen for the improved expression of gene targets in *Saccharomyces cerevisiae* is described that is based upon the activity of the luciferase from *Gaussia princeps.* Using the Unspecific Peroxygenase (UPO) from *Agrocybe aegerita* (*Aae*UPO) as a model protein, improvements in expression, effected through error-prone PCR-based mutation within the signal peptide (SP) domain, can be detected using fusion of the target to *Gaussia* luciferase encoded downstream of the first folded domain of the *Aae*UPO protein and luminescent assay of expression supernatants. In this way, previously undiscovered mutations within the SP of *Aae*UPO that improve expression were revealed, and then applied to the expression of full-length *Aae*UPO in *S. cerevisiae*. The system was validated against control expression constructs that were well or poorly expressed and indicated a 13.9-fold improvement in expression for the best mutant over the wild-type SP sequence. It is envisaged that this protocol may be applied generally to the high-throughput detection of improved expression in *S. cerevisiae* for constructs that are engineered using directed evolution techniques.

**Supplementary Information:**

The online version contains supplementary material available at 10.1038/s41598-025-09669-6.

## Introduction

The heterologous expression of recombinant proteins in prokaryotic and eukaryotic hosts is a key technique in biotechnology and pharmaceutical science for the production of biopharmaceuticals, enzymes and target proteins for various applications^1^. To meet this demand, it is essential that the production process is optimized, which includes addressing the protein-specific and host-specific bottlenecks encountered during in vivo protein production^2^. Eukaryotic hosts such as *Saccharomyces cerevisiae* and *Komagataella phaffii* (previously known as *Pichia pastoris*) are ideal for heterologous expression as they are easy to manipulate, have a short doubling time, and possess the intracellular compartments that permit post-translational modifications often required for the production of eukaryotic proteins^3,4^. An additional key feature of these eukaryotic hosts is that they permit the secretion of target recombinant proteins, although the production of these can often overwhelm the host organism.

In addition to the organelles that enable the proper folding of proteins and their post-translational modification, *S. cerevisiae* also possesses a well-established secretory pathway. Secreted proteins are equipped with a signal peptide^5^ (SP, e.g. the α-mating factor signal peptide^6,7^), which is essential for export of the target protein to the supernatant, and which is often co-opted for the expression and secretion of non-native protein targets. SPs are commonly 20–50 amino acids long, and despite their diversity, share an overall structure that is divided into three regions: an N-terminal region, a hydrophobic core or H-region, and a more polar C-terminal region^5,8^. In the first step of secretion, the SP interacts with the signal recognition particle (SRP)^9–11^a complex of proteins that recognises the SP and directs proteins to the secretory pathway. In addition to the SP, evidence has been presented suggesting that the amino acids immediately downstream of this region may also influence the cleavage, folding and maturation of the secreted protein^12,13^. These observations dictate that, when optimizing the expression of a target gene and the secretion of the product, the sequence of the SP, but also the identity of the residues downstream of it, should be considered when designing appropriate expression systems.

Signal peptide engineering is thus often used to tackle secretion challenges, such as protein aggregation before translocation to the ER or incorrect translocation^14,15^. Recent studies have also demonstrated the utility of machine learning (ML) techniques in identifying optimal signal peptides for the secretion of proteins, however, challenges remain with respect to the amount of laboratory screening necessary to validate the sequences that result from the experiments^16^.

Unspecific Peroxygenases (UPOs) are heme-thiolate enzymes that have been investigated for their potential as biocatalysts for selective oxygenation reactions^17–20^. UPOs originate from fungi, where they are thought to form part of the complement of enzymes secreted by these organisms for the purposes of lignin degradation. Given their industrial potential for applications in chemistry, much research has been dedicated to optimising the heterologous expression of these targets in *Saccharomyces* and *Pichia* for the purposes of high yielding, robust and reproducible protein production, some of which has focused on optimisation of the SP sequence. In pioneering studies by Alcalde and co-workers, directed evolution was performed on the gene encoding the UPO from *Agrocybe aegerita* (*Aae*UPO) to give a variant with improved activity, but also improved expression levels, in *S. cerevisiae*^21,22^. These improvements were attributed to, respectively, four amino acid substitutions in the SP region and five in the sequence of the mature protein. In a further study, Weissenborn and co-workers created a combinatorial library of UPOs and their SPs, and determined that, in some cases, the best heterologous expression of a UPO could be achieved using a non-cognate SP^23^. This was further demonstrated by our own successful expression of the ‘artificial’ peroxygenase artUPO in *Pichia pastoris* using the evolved SP from the *Aae*UPO gene^24^.

Each of these studies suggested a crucial role for the sequence of the SP region in the optimal heterologous expression of UPOs. Extensive exploration of this sequence for optimal expression may be best achieved using directed evolution methods, including error-prone PCR, as applied by Alcalde^21,22^coupled to a generic high-throughput plate-based screen that would report on the improved expression of the same target under the control of many evolved SP regions. In the work of Weissenborn, for example, high-throughput screening for expression was achieved through the inclusion of a GFP tag on evolved constructs, followed by validation through enzymatic assay^23^. Such a generic approach would have the advantage of its potential application to the optimisation of expression of many other interesting protein targets. In this study we combine a directed evolution approach to SP randomisation with a high-throughput luminescent screen that exploits the activity of the luciferase enzyme from *Gaussia princeps* (GLuc)^25^ to develop a platform for SP optimisation and validation. Random mutagenesis is applied to the SP region of *Aae*UPO constructs that contain a truncated protein target and GLuc encoded downstream. Following construction of the library and expression in *S. cerevisiae*, expression is detected by the luminescent signal emitted upon the application of GLuc reagents to the expression supernatant. In this way, a generic approach to SP optimisation has been developed, which is not dependent upon UPO activity, and hence has potential applications for the optimisation of expression through SP modification for a range of proteins.

## Results and discussion

### Design of the platform construct

**Fig. 1:**
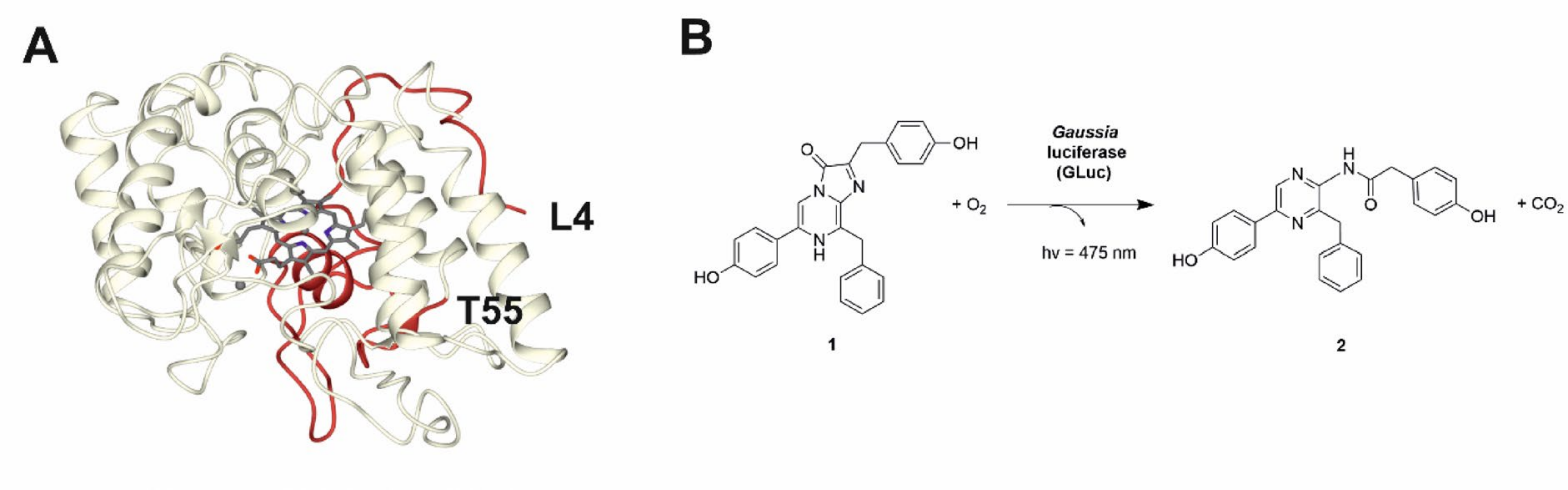
**A** Structure of *Aae*UPO-PaDa-I (PDB 2YOR showing truncated d55 fragment in red; **B** Oxidative decarboxylation of coelenterazine **1** to coelenteramide **2** catalyzed by GLuc.

In order to design the platform construct for the study, it was necessary to select the strain of *S. cerevisiae*, the heterologous host, a vector for transfection, a model SP-protein target system and a reporter gene. The *S. cerevisiae* strain INVSc1 was chosen, paired with the vector pESC-TRP for tryptophan auxotrophic selection. Based on our interest in UPOs we selected as the model SP-protein target the system that encodes the *Aae*UPO enzyme. Two SP variants were chosen: the wild-type sequence, which is native to *Agrocybe aegerita* (wt-*Aae*UPO)^26^ and the four-point variant (F12Y/A14V/R15G/A21D) used in the expression of *Aae*UPO-PaDa-I, (PaDa-I) which was developed by Alcalde and co-workers using directed evolution^21,22^. These would serve as negative and positive controls respectively for the expression of proteins under the control of native and mutated SPs, as the wt-*Aae*UPO SP was reported to yield poor expression of *Aae*UPO in *S. cerevisiae*, whereas the PaDa-I SP variant gave a 27-fold improvement^21,22^. For the protein sequence to be expressed under the control of the SPs, we chose as a model system a version of the mature *Aae*UPO protein truncated to the first folded domain of *Aae*UPO, which was selected upon analysis of the structure (PDB code 2YOR)^27^ to end 55 amino acids downstream of the SP region (Fig. 1A) and named ‘d55’. Single protein domains are often used independently to assess particular functions^28^ therefore we hypothesised that the first independently folded domain of the protein would serve as an adequate representative of the whole protein for the purposes of expression analysis under the selected signal peptides.

For the reporter gene, we chose the luciferase from *Gaussia princeps* (GLuc)^25^. This is a small (18.2 kDa) enzyme that catalyzes the oxidative decarboxylation of coelenterazine **1** (Fig. 1B) to coelenteramide **2**, in the process emitting light at a wavelength of 475 nm, giving an intense luminescence signal. This makes it an attractive reporter for use in high-throughput applications, permitting the rapid analysis of mutant library cassettes and the quick identification of promising variants^29,30^. Although the heterologous expression of GLuc has been achieved in *E. coli* using a solubility enhancement peptide tag^31^ insect cells^32,33^cell-free systems^34^ and in the yeast *Kluyveromyces marxianus*, following extensive signal peptide analysis^35^to our knowledge, no successful expression in *S. cerevisiae* has previously been reported.

### Validation of the GLuc ssay

The GLuc assay was to be validated using two constructs, the first construct contained the wt-*Aae*UPO SP; the second contained the evolved PaDa-I SP, each coupled to the *Aae*UPO-d55 protein truncate, and were named ‘n55-Gluc’ and ‘m55-GLuc’ respectively (Supplementary FigureS1). As protein produced under the control of the wild-type and evolved SPs were reported to give very poor and very good expression respectively in *S. cerevisiae*^21,22^it was expected that expression of these constructs when ligated to GLuc would give poor and good signals respectively.

The constructs n55-GLuc and m55-GLuc were built using In-Fusion cloning in the plasmid pESC-TRP and recovered from *E. coli*. Cloning was confirmed by restriction enzyme digestion and sequencing of isolated plasmid DNA. *S. cerevisiae* was transformed with the plasmids and successful transformation colonies were grown on a 50 mL scale and induced with galactose. Following this, samples were removed at 8 and 24 h for analysis of supernatants using the GLuc assay, which was conducted using 96-well plates with analysis on a luminometer at 475 nm (see Methods).

**Fig. 2:**
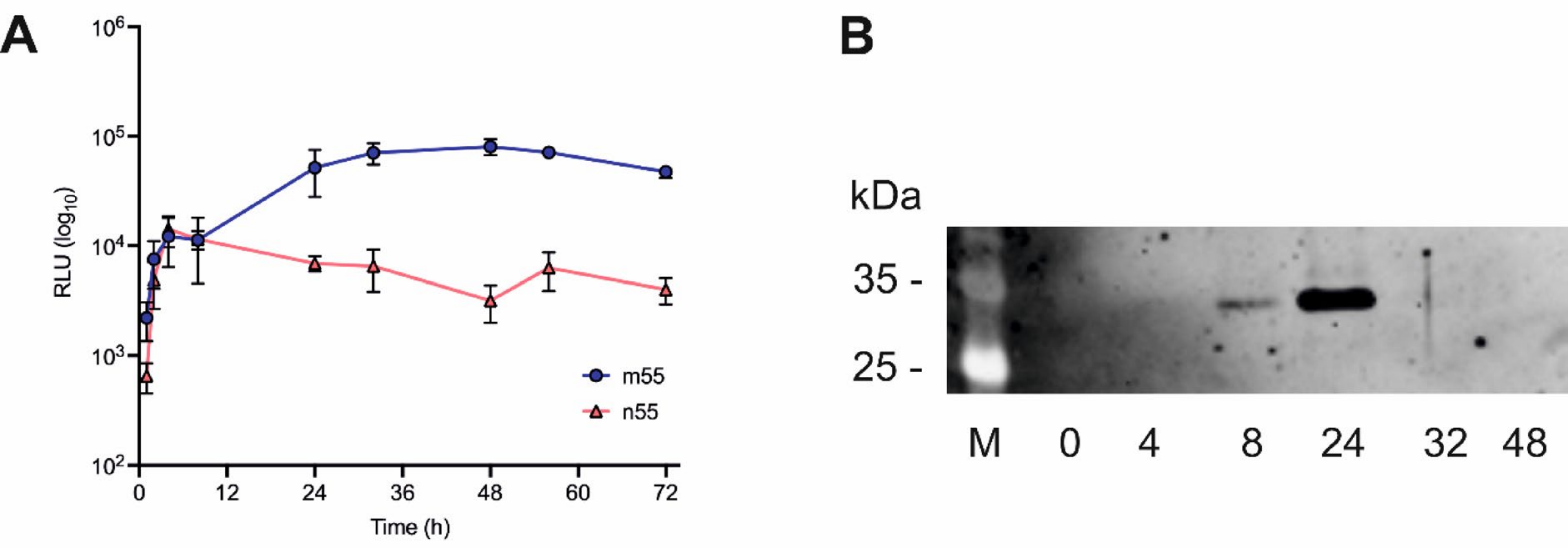
**A** Comparison of the signal detected for n55-Gluc (orange) and m55-Gluc (blue line) expressed in shake flasks. RLU is Relative Light Units. m55-Gluc gave a 7.5-fold improved signal at 24 h compared to n55-Gluc. Data are presented as RLU mean +/- SEM (*n* = 3). **B** The peak in protein expression for m55-GLuc was identified to be at around 24 h from western blot analysis (the complete blot can be found in the Supporting Information Figure S2).

A comparison of the signal evolved over time using n55-Gluc and m55-Gluc (Fig. 2A) showed that, at 24 h of protein production the signal produced from m55 was 7.5 times higher than that produced from n55, indicating a much higher level of gene expression under the control of the PaDa-I SP than under the wt-*Aae*UPO SP. Furthermore, the presence of the protein was validated by Western blot analysis (Fig. 2B), indicating maximal expression at around the 24 h time point. These results accord with the previous report of significantly improved expression using the evolved SP^21,22^ and validate the GLuc approach as a useful assay for gene expression using different SPs.

**Fig. 3:**
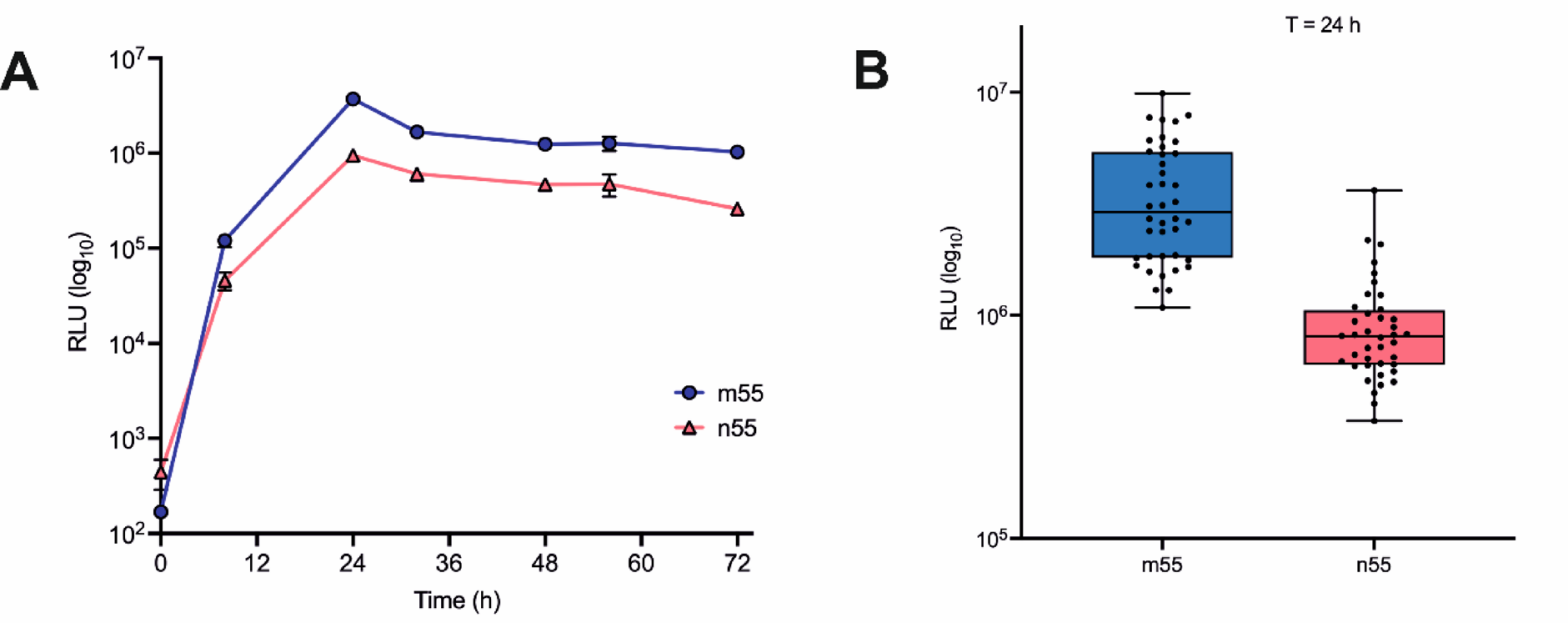
**A** Expression and assay of n55-GLuc (orange) and m55-GLuc (blue) in 96-well plates. The peak in signal occurred at 24 h expression. **B** 24 h signal RLU distribution of m55 and n55 (log_10_ scale). The plot shows an average 5-fold improvement in signal for m55-GLuc compared to n55-GLuc. Data are presented as RLU mean +/- SEM (*n* = 44).

With consideration for future higher-throughput assays that would test evolved SP libraries, gene expression was then tested in 96-well plates and optimized using 44 colonies each of the two model constructs n55-Gluc and m55-Gluc. Supernatant samples were then collected at 8, 24, 48, 56 and 72 h and subjected to the GLuc assay. In each case the luminescence signal was measured at 1 s intervals for the first 10 s of the reaction (Fig. 3A).

In the 96-well plate, both n55-GLuc and m55-GLuc displayed a peak in GLuc assay response at 24 h, indicative of peak expression at around this time. In addition, a study of the distribution of signals for all colonies showed a 5-fold higher signal for m55-GLuc compared to n55-GLuc at the 24 h time point (Fig. 3B). Taken together, these preliminary analyses showed that the GLuc reporter assay should indeed be suitable for the high-throughput and rapid assay of evolved SP libraries, with the “flash” kinetic properties of GLuc, coupled with the use of an automatic injection plate-reader, permitting samples in a 96-well plate to be analysed in under 15 min.

### Directed evolution of the SP

**Fig. 4:**
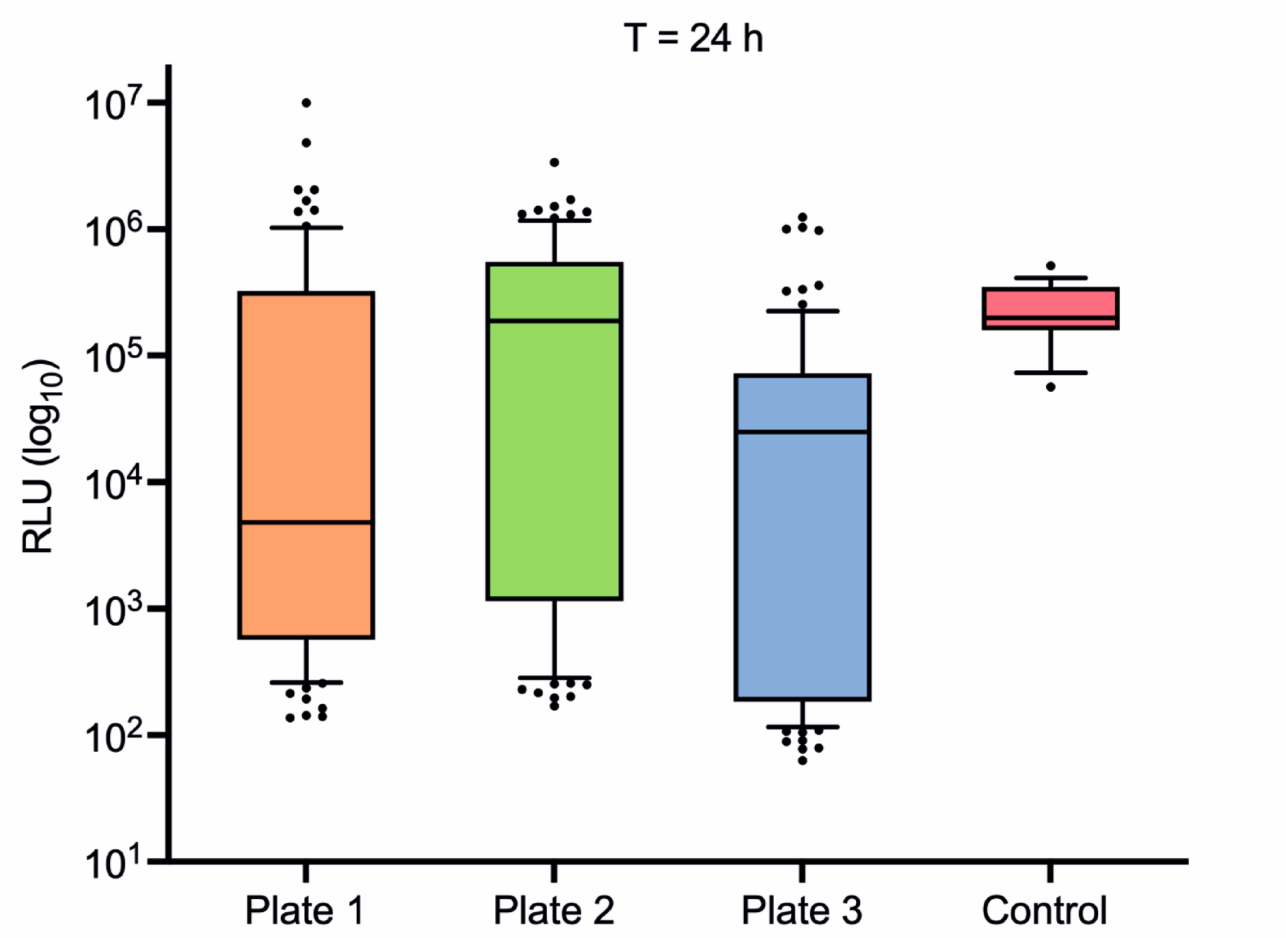
GLuc HTP assay of 264 mutant colonies at 24 h after induction. The cultures were divided into three plates with 88 colonies each. Outliers with a signal higher than the control were identified and selected for sequencing. Data are presented as RLU mean +/- SEM (*n* = 3).

The directed evolution of the SP required two main steps: First, the diversification of the genetic sequence of interest by random mutagenesis using error-prone PCR (epPCR), and then the screening and selection of mutant variants with the desired characteristic through the GLuc high-throughput (HTP) assay. The genetic element of interest was the SP of *Aae*UPO, which was diversified by error-prone PCR, with the technique optimized for the short SP sequence of 129 bp. n55-GLuc was used as the starting construct, and the SP was subjected to mutagenesis as described in the Methods. The vector containing n55-GLuc was linearised by inverse high-fidelity PCR, and the products (vector and SP library generated by epPCR) were purified and used to transform *S. cerevisiae*, in a mixture of 2:1 ratio for the SP library product and linear vector respectively. The genetic machinery and high-rate of homologous recombination in *S. cerevisiae* permits the reconstitution of episomal plasmids in vivo, as long as an overlapping region of 40–50 bp is included between the linear plasmid and the insert, at the site of homologous recombination^36,37^. Plasmids were reconstituted in vivo, resulting in colonies containing different SPs derived from the mutagenized library. Transformants were picked and inoculated into 96 deep well plates, after which gene expression was performed and the supernatants were assayed using the GLuc HTP assay (Supplementary Figure S3). A total of 264 mutant colonies was tested simultaneously in 3 × 96 well plates, each of which included 88 mutant colonies and 8 of n55-GLuc as controls (Fig. 4).

The results shown in Fig. 4 showed that the random mutagenesis of the SP created considerable variability in response in the library analysed, with signals ranging from undetectable to out-of-range. Lower variability was observed in the control samples, with recorded signals between 10^5^ and 10^6^ RLU. Fifteen sample outliers were selected for sequencing, each named for their grid location on Plates 1, 2 or 3. These were 1C1, 1D02, 1B02, 1G01, 1F04, 1B04, 2G10, 2H06, 2F2, 2H03 and 3C10, 3C11, 3E11, 3B10 and 3D6. Of the 15 sequenced samples, three were identified that contained mutations in the SP sequences. The remainder displayed the native sequence and were considered to be false positives^21,22^. Mutant 1C1 had Y22N; mutant 2F2 had V11A, F12I and A21T; and mutant 3D6 had R15G, A18T and Q30L.

**Fig. 5:**
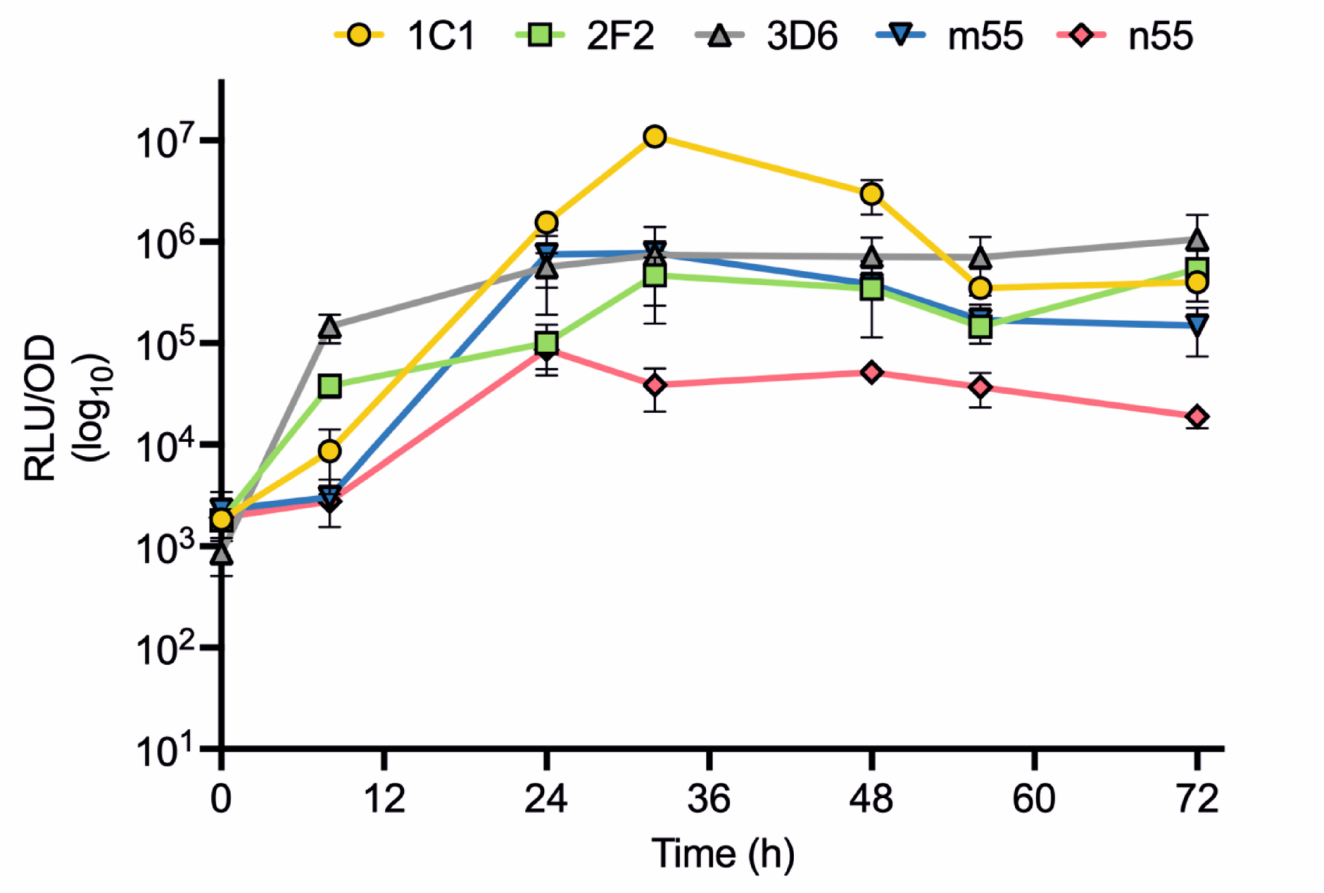
Expression and assay of constructs featuring SPs with mutations in novel positions: 1C1, 2F2 and 3D6, compared with n55 and m55. In this case the peak response recorded was for samples taken at 32 h after induction. The results show a 1092-fold higher signal for the 1C1 variant compared to n55-GLuc. Data are presented as RLU mean +/- SEM (*n* = 3*).*

In order to validate the observations of improved expression using the novel SP mutants, the growth of selected colonies was then scaled up to 50 mL. Samples of supernatant were then collected at time intervals and evaluated for expression using the GLuc assay (Fig. 5).

**Fig. 6:**
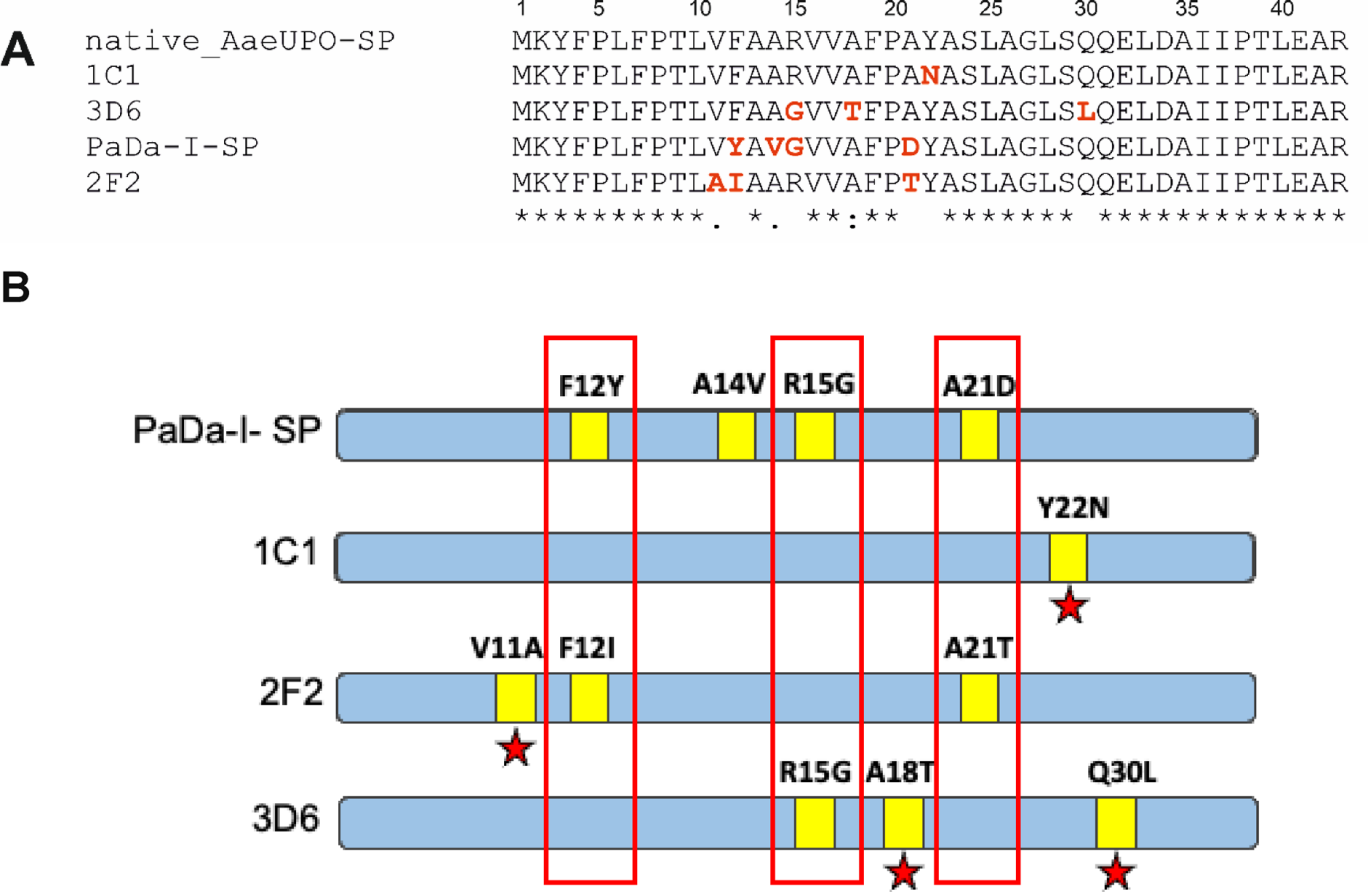
**A** Sequence alignment of native, PaDa-I and mutated SPs; **B** Highlighted mutations found on the mutated SPs 1C1, 2F2 and 3D6 compared to the evolved PaDa-I-SP sequence. Mutation sites identified at positions 12, 15 and 21, are also present in PaDa-I-SP. Mutations highlighted by red stars have been identified in new amino acid positions.

The constructs displaying the most improved activities were sequenced and the composition of their SPs were then compared with that of the PaDa-I SP included in the m55-GLuc construct (Fig. 6A and B). The comparison revealed a number of mutations in improved variants, including those at amino acid sequence positions 12, 15 and 21, which were in the same loci as those in the PaDa-I SP. As with PaDa-I SP, most of these and other mutations occurred within the hydrophobic core region of the SP (V11A, F12I, R15G, A18T), while three (A21T, Y22N, Q30L) were located between the C-terminal region and the PP (pro-peptide) sequence.

Of the mutations in the hydrophobic core of the SP, two (V11A and F12I) do not cause substantial changes in the chemical character of the amino acid side chain, appearing to confirm the importance of retaining hydrophobic side chains in these positions for functional secretion. Two others (A18T and A21T) increase the hydrophilicity, while one (R15G) reduces it. Previous analyses suggest that a conserved sequence motif of A-X-A may precede the SP cleavage site^38^. Analysis with the SignalP6.0 server^39^ suggests that this site may be A^21^YA in the wt-*Aae*UPO sequence (Supplementary Figure S4). Hence, mutations A21T and Y22N in variants 2F2 and 1C1-respectively may have an influence on cleavage of the SP in these constructs. The signal sequence 1C1 was analysed using the SignalP6.0 server and compared with the native signal peptide sequence. The results suggest that the cleavage of 1C1 occurs at the A^21^XA motif, while for the native signal peptide, it is unclear if cleavage would occur at V^18^XA or A^21^XA. In the literature it has also been reported that amino acids in proximity to the cleavage site are small and flexible, so as not to interfere sterically with the cleavage of the SP^40^. It is possible therefore that the Y22N mutation may have a beneficial effect on secretion by removing steric hindrance close to the cleavage site.

The reproducibility of the results obtained from the SP mutant library screens confirmed that the mutant giving the highest GLuc assay signal, 1C1, gave a signal at least 10-fold higher than the other variants from the experiment and 1092-fold higher than that of n55-GLuc. Mutant 1C1-GLuc was thus identified as having the ideal SP variant with which to further analyse secretion of the full-length target protein *Aae*UPO.

### Evaluation of evolved SPs using expression of full length AaeUPO

In order to evaluate the effectiveness of the SP mutations in a working system, the SP from mutant 1C1 was then applied to the expression of full length *Aae*UPO-PaDa-I and compared with the expression of the same gene under the control of wt-*Aae*UPO SP and PaDa-I SP as negative and positive controls respectively. For these experiments the sequence encoding the *Gaussia* luciferase gene was now removed, and each variant SP sequence was fused upstream of the *Aae*UPO-PaDa-I mature protein sequence to create constructs pESC/1C1-PaDa-I (developed in this study), pESC/PaDa-SP-PaDa-I^17,18^ and pESC/native-SP-PaDa-I (Supplementary Figure S5).

**Fig. 7:**
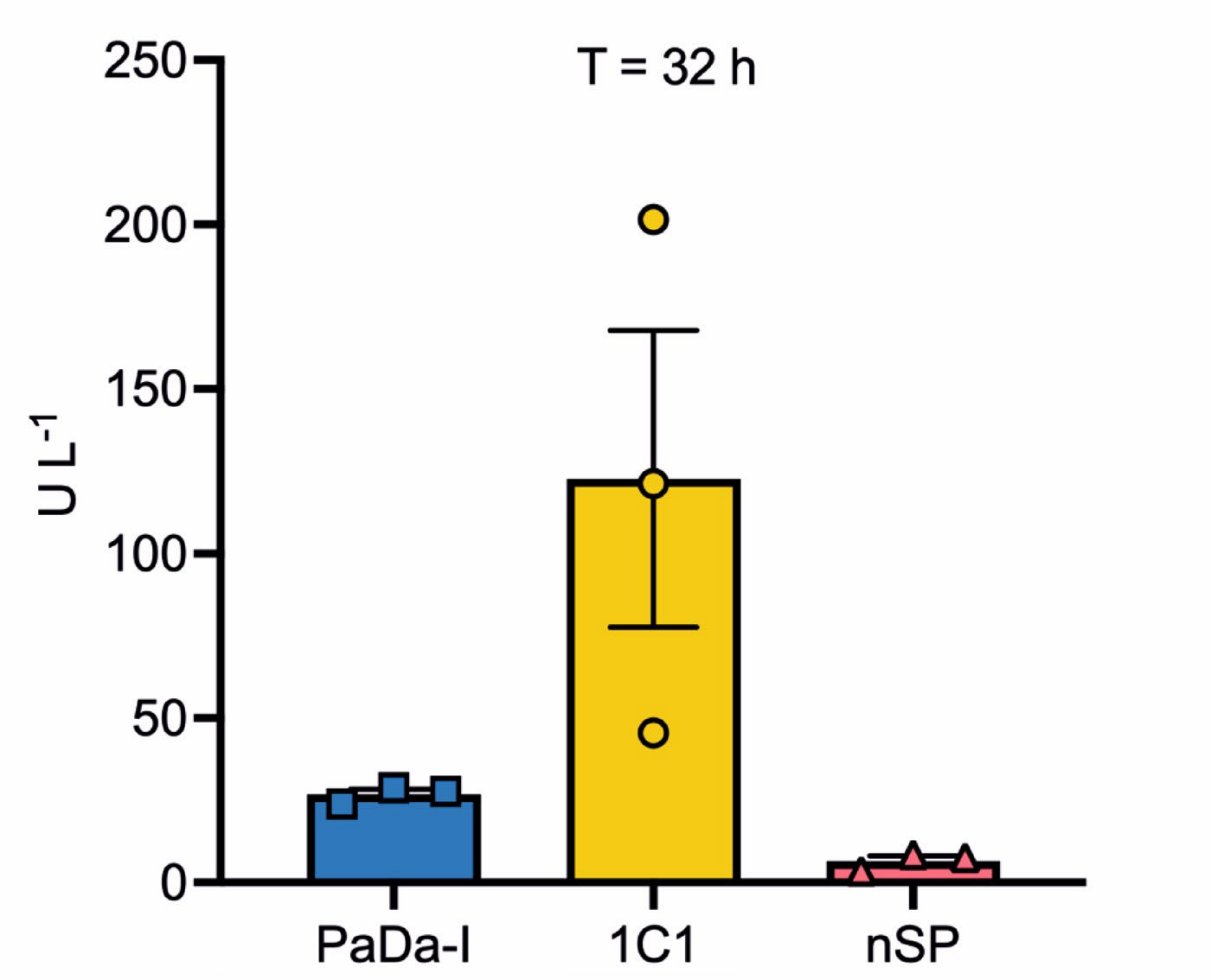
Peroxygenase activity of *Aae*UPO-PaDa-I enzymes produced using PaDa-I-SP, 1C1-SP and wt-*Aae*UPO (n-SP) signal peptides, measured using the NBD assay. Data are presented as RLU mean +/- SEM (*n* = 3).

Cells of *S. cerevisiae* were transformed with the linearised vectors and gene expression was then performed on a 50 mL scale. Peroxygenase activities of the supernatants were determined using the established UPO activity assay that monitors the transformation of nitrobenzoxadiazole (NBD). As the mature *Aae*UPO-PaDa-I protein produced should be identical in all three cases, it was reasoned that these analyses would permit a better understanding of which of the different SPs gave the best expression of the target. Analyses were performed with three biological replicates of each construct (Fig. 7).

The results showed that for supernatants derived from 32 h growth, all the replicates using the 1C1-SP displayed significantly higher activity than those using either PaDa-I-SP or n-SP. Replicate 1C1-1, for example exhibited 7-fold greater activity compared to replicate PaDa-I-3, the best performing replicate out of all of the other clones. With consideration for the variability in activity of the 1C1-SP replicates, a one-way Analysis of Variance (Anova) test was performed on the measurements determined at 32 h for the PaDa-I-SP, n-SP and 1C1-SP *Aae*UPO-PaDa-I constructs, giving a p-value of 0.04, confirming the statistical significance of the measured activities. It is not unusual to register heterogeneity between clones, and the reason may vary, from the difference in plasmid copy number^41^to variation in the inoculum due to differences in the growth rate of starter cultures.

Overall, the SP in the 1C1 variant improved secretion of *Aae*UPO by a factor of 13.9-fold compared to either the native SP, with supernatants derived from clones using the 1C1-SP giving average activities in the NBD assay of 122.7 U L^−1^_,_ compared to those using the native SP of 8.8 U L^−1^. Even the 1C1 clone with the lowest activity (1C1-3, 45.4 U L^−1^) outperformed any nSP replicates. 1C1 clones also routinely out-performed those using the established PaDa-I SP with an average improvement of 5.3-fold in NBD activity, with the highest activity measured at 28.8 U L^−1^. These results are congruent with expression levels measured using the GLuc assay on the truncated UPO d55, where it was observed that d55 expressed under the control of the 1C1-SP displayed a 110-fold higher luminescence signal than those using either the PaDa-I-SP or nSP. The validation with the full-length *Aae*UPO also suggests that the first folded domain of the protein d55 may indeed effectively act as a sufficient representative fragment for the intact enzyme in assay development.

## Conclusion

The heterologous production of proteins for biomedical or biotechnological applications requires that high yielding systems are established to optimise the yields of the proteins of interest. In this regard, the development of high throughput plate-based screens for expression optimisation are extremely valuable. In this study, we have shown that the fusion of the luciferase from *Gaussia princeps* to targets of interest permits a rapid and high throughput evaluation of the expression of a protein target under the control of mutant signal peptides obtained using random mutagenesis. The model system has been validated with preparative expression tests using full length proteins under the control of native and mutant SPs with good agreement. Although the wider applicability of the screen awaits more comprehensive testing against other proteins, it is envisaged that the GLuc screen may be suitable for application to the expression optimisation of a range of targets in *Saccharomyces*, with potential applications to other widely used yeast hosts, such as *Komagataella phaffii*, in which the efficiency and utility of the system also remain to be evaluated.

## Methods

### Chemicals and restriction enzymes

Coelenterazine was purchased from (Cambridge Bioscience, UK) and NBD from Sigma-Aldrich Company Ltd. (Dorset, UK). Restriction enzymes were purchased from New England Biolabs (Ipswich, UK).

### Cells and cultivation media

*E. coli* stellar competent cells were purchased from Takara Bio Europe Clontech (St Germain-en-Laye, France). *S. cerevisiae* InvSc1 strain was purchased from Invitrogen (ThermoFisher, UK). For *E. coli* growth, LB was purchased from Merck Chemicals Ltd. (Nottingham, UK). For *S. cerevisiae* growth SC-trp dropout medium from Formedium (Hunstanton, GB) was used. Yeast Extract Peptone was purchased from Merck Chemicals Ltd. (Nottingham, UK). YNB was obtained from Sigma-Aldrich Company Ltd. (Dorset, UK).

### Oligonucleotides and genes

Oligonucleotides were purchased in the lowest purification grade “desalted” and minimal quantity from IDT Integrated DNA Technologies (Leuven, Belgium). *Aae*UPO variant genes were previously generated in our laboratory^42^. The nSP-*Aae*UPO-d55 truncated gene was purchased from Genescript (Oxford, UK).

### Expression plasmid construction for *S. cerevisiae*

The vector pESC-TRP was used as the backbone structure for expression in *S. cerevisiae* and for propagation in *E. coli*. It enables antibiotic resistance (ampicillin) and yeast autroxophy selection (trp1-289 marker). To enable gene expression the GAL1,10 promoter was used. The vector was digested with BamHI and XhoI for gene integration. *Gaussia luciferase* (GLuc) was initially integrated in the MCS (multiple cloning site) downstream of the GAL1,10 promoter. The truncated gene *Aae*UPO-d55 with the nSP and PaDa-I SP were cloned at the N-terminal of GLuc and downstream of the GAL1,10 promoter. The constructed plasmids were named n55-GLuc and m55-GLuc and used for the expression of the *Aae*UPO-d55 truncate under the native and PaDa-I SPs respectively. For the construction of the plasmids pESC/1C1-PaDa-I, pESC/native-SP-PaDa-I and pESC/PaDa-SP-PaDa-I, the respective plasmids with the truncated *Aae*UPO-d55 gene were used as the backbone and linearized by inverse PCR to delete the sequence *Aae*UPO-d55-GLuc. The *Aae*UPO-PaDa-I gene variant without the SP was amplified by PCR and cloned into the three plasmids downstream of the respective SPs.

### Error-prone PCR and plasmid reconstitution in vivo

The plasmid n55-GLuc was used as the backbone for the epPCR experiments and for plasmid reconstitution in vivo in *S. cerevisiae*. The vector was linearised by High-Fidelity PCR to delete the native signal peptide sequence and it was used during the epPCR experiments as the donor of the native SP sequence. The epPCR experiments were performed with the forward and reverse primers 20 bp upstream and downstream of the signal peptide. This created a 40 bp region overlap between the mutated signal peptide and the linear vector, necessary for in vivo reconstitution of the plasmid in *S. cerevisiae*. The epPCR reaction was optimised for small DNA fragments (the SP sequence is 129 bp). The reaction was set up in a final volume of 100 µL, containing Tris 10 mM (pH 8.3), KCl 50 mM, MgCl_2_ 12 mM, dATPs and dGTPs 0.2 mM, dCTPs and dTTPs 1 mM, ep-SP-F and ep-SP-R 0.4 µM, MnCl_2_ 0.2 mM, template DNA (nSP-GLuc) 0.02 ng, *Taq* polymerase 0.1 U µL^−1^. The thermocycler was set with the following parameters: 94 °C for 30 s, 18 cycles of 94 °C for 1 min, 60 °C for 1 min, 72 °C for 3 min, followed by a last extension step of 72 °C for 5 min. The epPCR product and the linear plasmid were then used to transform *S. cerevisiae* in a 2:1 ratio respectively (300 ng to 150 ng respectively) to give a final DNA concentration of 450 ng.

### Microculturing gene expression in *S. cerevisiae*

 Single colonies were picked and inoculated in a sterile 96 deep-well plate, containing 250 µL of YNB-TRP medium with 2% (w/v) raffinose. Plates were incubated at 30 °C for 48 h, shaking at 900 rpm to avoid sedimentation. 1 mL of YNB-TRP medium with 2% (w/v) galactose was added to each well to induce gene expression. The culture plates were incubated at 30 °C for 48–72 h, with shaking at 900 rpm. Supernatant samples were collected at selected time points (T = 0 h before induction, 8 h, 24 h, 32 h, 48 h and 56 h after induction) by transferring 50 µL of media to a new round bottom 96 well plate. 5 µL of the samples were used for OD_600_ measurement; the remaining 45 µL was centrifuged at 2800 rpm at 4 °C for 10 min (Sigma 3–16 KL, Sigma) and the supernatant was transferred to a new 96 well plate. The plate was then stored at −80 °C, or the samples were diluted prior being analysed through the *Gaussia* luciferase high-throughput assay.

### Shake flask expression in *S. cerevisiae*

One colony was inoculated in 10 mL of YNB-TRP medium with 2% (w/v) raffinose and incubated for 24–48 h at 30 °C with agitation at 230 rpm. A T = 0 h sample was collected and, to induce gene expression, the amount of culture necessary to have OD_600_ = 0.4 in a final volume of 50 mL was centrifuged at 1500 g for 5 min at 4 °C. The supernatant was discarded and the cell pellet resuspended in 50 mL of YNB-TRP medium with 2% (w/v) galactose. The culture was incubated at 30 °C with agitation at 230 rpm. Culture samples were collected at selected time points after induction in a 1 mL volume. Samples were centrifuged at 17,900 *g* for 1 min, and supernatant was collected and stored at −80 °C prior to GLuc assay analysis.

### Western blot analysis

SDS-PAGE 12% gels were run with the supernatant samples collected at time intervals during expression, using as a marker PageRuler™ Plus Prestained Protein Ladder, 10 to 250 kDa (ThermoFisher scientific, UK). Protein transfer was performed with Invitrogen iblot 2 dry blotting system set at 20 V and 1 Amp for 6 min. The nitrocellulose membrane was washed with blocking buffer PBST 3% milk (PBS, Tween-20 0.1%, 3% milk) and then blocked for 3 h with gentle shaking at room temperature. The membrane was then incubated overnight in blocking buffer containing 1:1500 dNGLuc monoclonal primary Ab (Proteintech, UK). Following overnight incubation, the membrane was washed repeatedly with PBST 3% milk, prior a second incubation for 1 h, with blocking buffer containing 1:80,000 anti-mouse IgG Peroxidase antibody produced in rabbit (Sigma, UK). Afterwards, the membrane was washed repeatedly with PBS buffer, and then visualized on iBright Invitrogen imager following 5 min incubation at RT with ECL (Invitrogen) chemiluminescent solution.

### Gaussia luciferase assay

The *Gaussia* luciferase high-throughput (HTP) assay was performed with an auto-injector luminometer (Clariostar, BMG Labtech) set to record at 475 nm. A buffered solution of PBS containing 5 mM NaCl at pH 7.2 was prepared containing 0.05% (v/v) 100X coelenterazine. The assay was performed using white, flat-bottomed 96 well plates containing 20 µL of 20 times diluted supernatant samples. The automatic plate reader was primed with the coelenterazine buffered solution and the program was set to automatically inject 50 µL of the solution to the supernatant samples. The gain was set at 3500 and the settings were set to take one measurement every second for the first 10 s of the reaction, starting from 2 s after injection.

### 96-well plate cultivation and assay

Cells were grown in two 96 well plates in 250 µL YPD 2% (w/v) raffinose for 24 h. Induction of gene expression was then performed by adding 1 mL of YPD 2% (w/v) galactose, and supernatant samples were then collected at 8, 24, 48 and 56 h. The GLuc assay was performed using a Clariostar auto-injector plate reader and a luminescence signal was measured at 1 s intervals for the first 10 s of the reaction.

### NBD assay

The reaction screening was performed in a 1 mL UV quartz cuvette (1 cm) containing KPi buffer 50 mM, pH 7.0, 1 mM NBD, 75 µL of crude supernatant sample and 2 mM H_2_O_2_. The absorbance was measured at 420 nm over the duration of 2 min. To measure activity (U (µmol min^−1^)), the change in absorbance was plotted against time and the reaction rate (*k*_obs_) was determined against the NBD extinction coefficient (ε_420_ (M^−1^ cm^−1^) = 9700), using the equation below.

## Electronic supplementary material

Below is the link to the electronic supplementary material.

Supplementary Material 1

## Data Availability

The data that support the findings of this study are available from the corresponding authors (JC and GG) upon reasonable request. The sequences generated during the current study are available from NIH Genetic Sequence Database Genbank with the following accession numbers: BankIt 2902982 1C1 PQ729992; BankIt2902982 2F2 PQ729993; BankIt2902982; 3D6 PQ729994; BankIt2902982 n55 PQ729995; BankIt2902982 m55 PQ729996; BankIt2902994 nSP PQ729997; BankIt2902994 PaDa-SP PQ729998; BankIt2902994 1C1-SP PQ729999.
