# Supplementary material for: Identification of improved signal peptides for heterologous expression in Saccharomyces using a screen that exploits Gaussia luciferase
Source: Sci Rep. 2025 Jul 4;15:23962. doi: 10.1038/s41598-025-09669-6 (PMC12227527; doi:10.1038/s41598-025-09669-6)
Supplement: Supplementary file 1 — Supplementary Material 1 [file 41598_2025_9669_MOESM1_ESM.pdf]

Identification of Improved Signal Peptides for Heterologous Expression in *Saccharomyces* using a Screen that Exploits *Gaussia* Luciferase

Ginevra Camboni,[a,b] Jared Cartwright[b]* and Gideon Grogan[a]*

Departments of Chemistry[a] and Biology,[b] University of York, Heslington, York YO10 5DD U.K.

SUPPORTING INFORMATION

1. Sequences of Primers

Table S1: Primer sequences used in this study.

**Name**	**Sequence (5' – 3')**	**Description**
>AaeUPO-nSP-F	TAATTATTCGAAACGATGAAATATTTTCCCCTGTTCCCAAC	N-t of *Aae*UPO native signal peptide- forward amplification.
>AaeUPO-nSP-R	AGGTAATCCTGGCTCTCGGGCCTCGAGTGTTGG	C-t of *Aae*UPO native signal peptide- reverse amplification.
>m55 R	ATTCTCGGTAGGTTTGGTTGCAACGCCATTTCTCGGGAG	m55 truncation of *Aae*UPO- reverse amplification.
>DF	ACCCCGGATCCAAAAATGAAATATTTTCCCCTGTTCCCAACCTTGG	*Aae*UPO N-terminal. Tail to clone in linear pESC-TRP/GLuc.
>ep-SP-F	GTTAATATACCTCTATACTTTAACGTCAAG	Amplification of nSP for epPCR, complementarity to the n55 vector upstream (ep-SP-F) and downstream (ep-SP-R) of signal peptide.
>ep-SP-R	CAGAGCTATTCTCGAGAGGA	
>GC001_2 F	AAACCTACCGAGAATAATGAAGACTTCAATATC	pESC linearizarion and GLuc SP deletion by inverse PCR..
>GC001_2 R	TTTTGGATCCGGGGTTTTTTC	pESC linearization by inverse PCR.
>GC001-1F (GLuc_F)	AAAAAACCCCGGATCCAAAAATGGGTGTGAAGGTTTTGTTCGC	GLuc gene amplification. Tail for cloning in pESC-TRP.
>GC001-1R (GLuc_R)	ACCAAGCTTACTCGAGTTAGTCACCACCCGCGCCTTTAATTTTG	
>HF-GC001-F	GAGCCAGGATTACCTCCTGG	Linearization of n55 vector, for signal peptide deletion.
>HF-GC001-R	TTTTGGATCCGGGGTTTTTTCTC	

2. Plasmid maps for truncated sequences

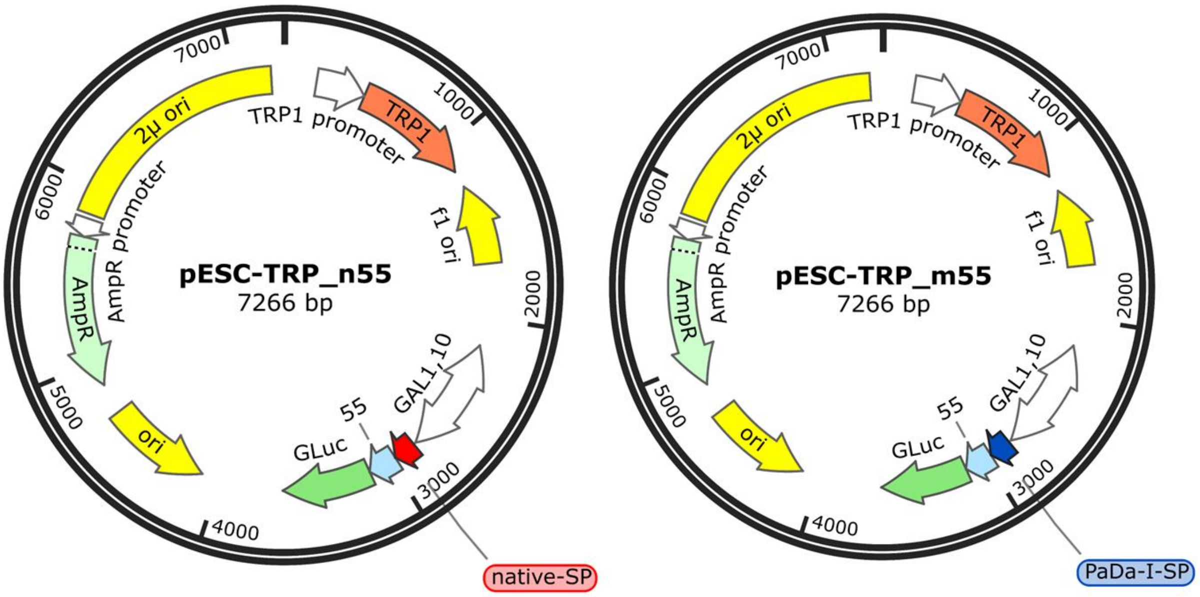

n55-GLuc **m55-GLuc**

Supplementary Figure S1: n55-Gluc and **m55-Gluc** constructs used for the analysis of protein production under the wt-*Aae*UPO and PaDa-I SPs respectively.

3. Western blot

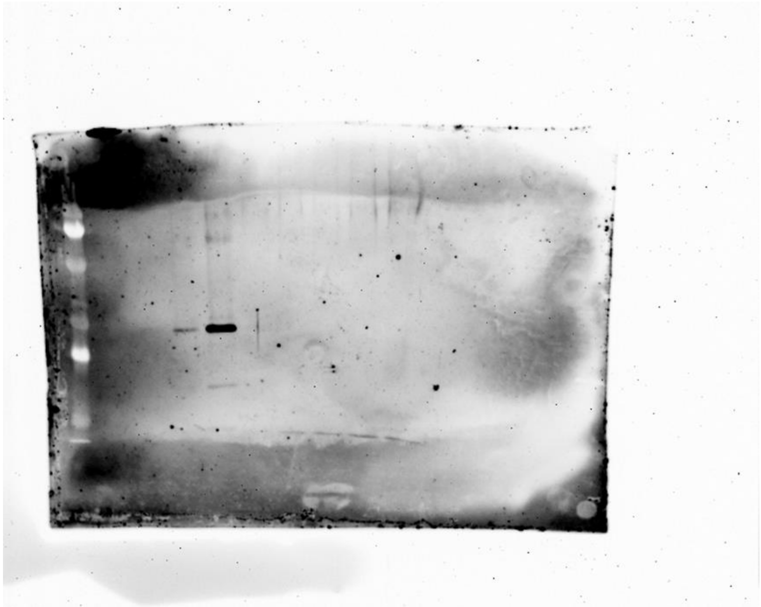

Supplementary Figure S2: Complete western blot, detail of which is shown in **Figure 2B**.

4. Workflow for Directed Evolution and Assay

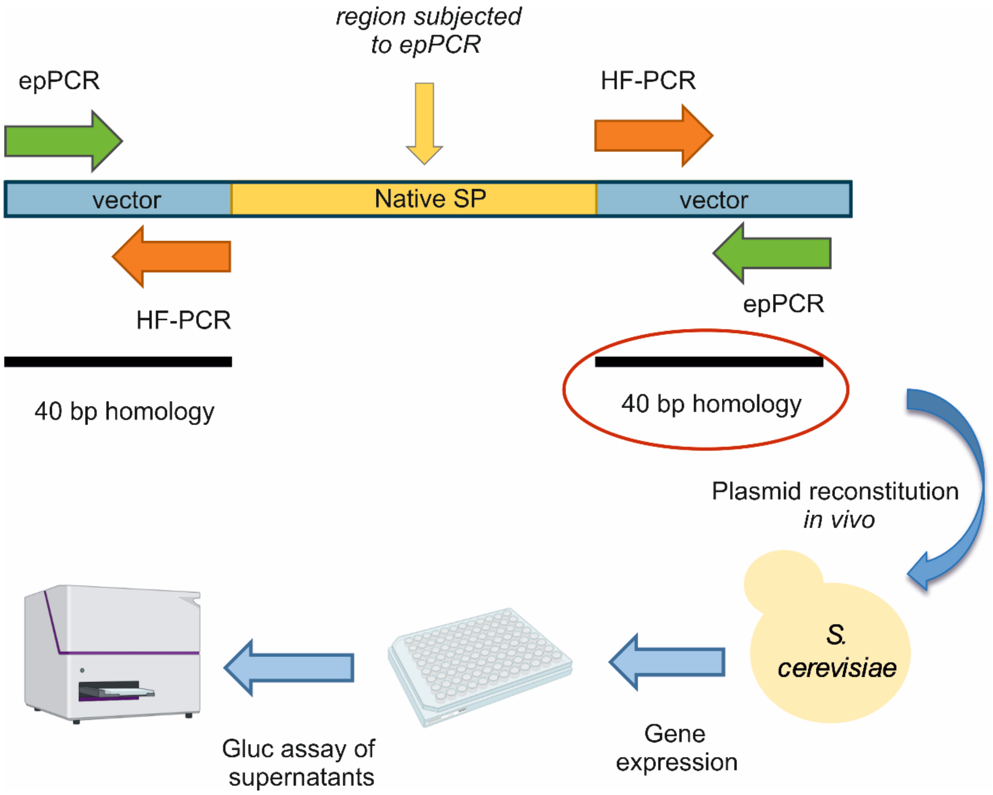

Supplementary Figure S3. Schematic illustrating epPCR of SP in n55-GLuc, high-fidelity PCR (HF-PCR) of the vector, transformation of *S. cerevisiae*, gene expression and supernatant analysis using the GLuc HTP assay. The native signal peptide sequence was subjected to epPCR, while the vector was amplified by HF-PCR. Between the two regions there was a minimum of 40 bp overlap, which is necessary for *S. cerevisiae* to reconstitute the plasmid *in vivo.*

5. Analysis of Signal Peptide Sequences using SignalP 6.0.

A) 1C1

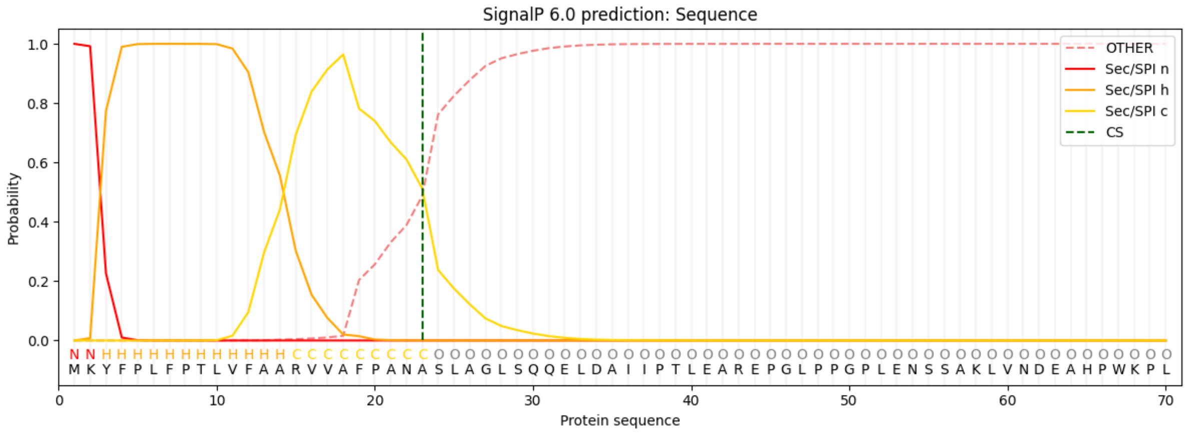

B) nSP

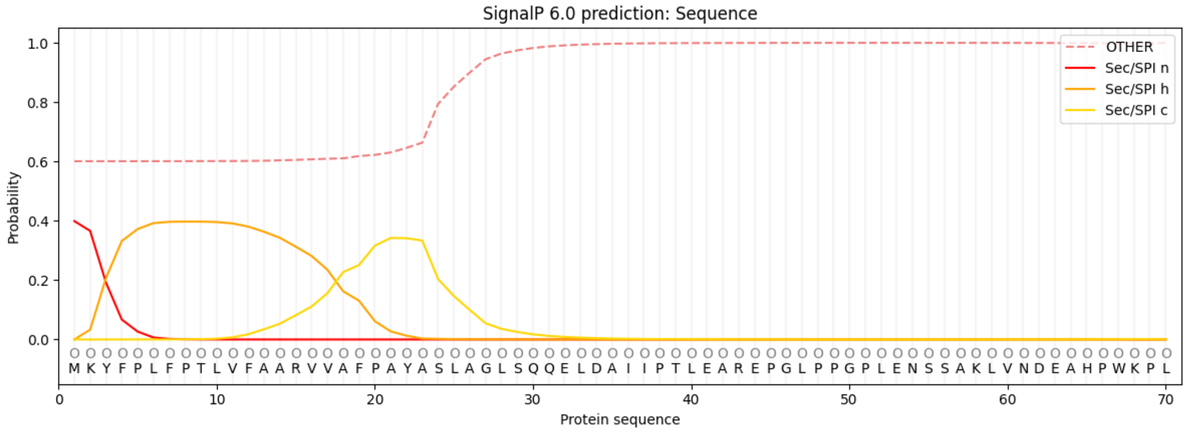

Supplementary Figure S4: A) The sequence of 1C1 was analyzed with the SignalP6.0 server, which predicted the cleavage site to be at the A^{21}XA site with 0.99 confidence. **B**) The analysis of the nSP sequence through the SignalP6.0 did not predict a cleavage site.

6. Plasmid maps for full length sequences

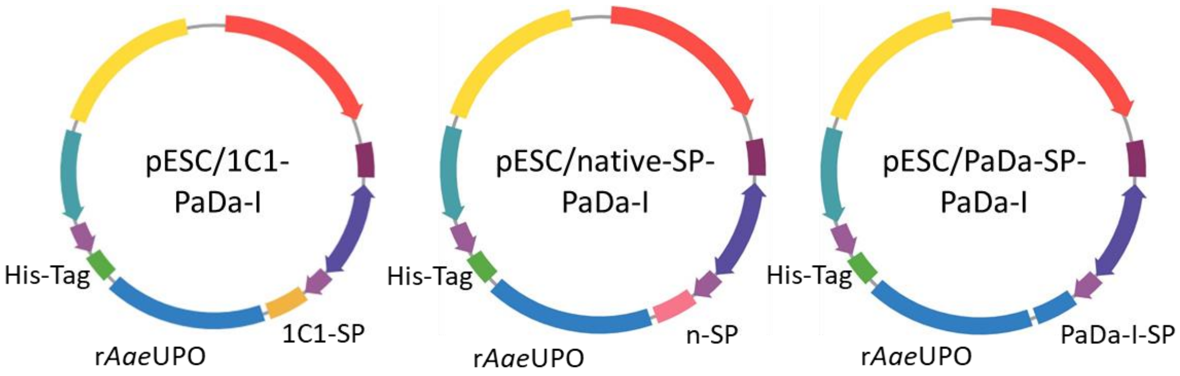

Supplementary Figure S5. Constructs used for comparing the expression of full-length *Aae*UPO-PaDa-I with 1C1, wt-*Aae*UPO (native, n-SP) and PaDa-I SPs.
